# Ten-Year Experience with Open Prostatectomy in Maiduguri 

**DOI:** 10.5402/2012/406872

**Published:** 2012-11-27

**Authors:** Ibrahim Ahmed Gadam, Ali Nuhu, Suleiman Aliyu

**Affiliations:** ^1^Department of Surgery, University of Maiduguri Teaching Hospital, PMB 1414, Borno State, Maiduguri, Nigeria; ^2^Department of Surgery, College of Medical Sciences, University of Maiduguri, Nigeria

## Abstract

*Background.* Benign prostatic hyperplasia is the most common cause of lower urinary tract obstruction in the elderly male. *Aim.* To evaluate the effectiveness, safety, and outcome of open prostatectomy in a Nigerian teaching hospital. *Material and Methods.* Two hundred and fifty-three men with lower urinary tract obstruction clinically due to benign prostatic hyperplasia (BPH) underwent open prostatectomy over a ten-year period (January 2001–December 2010). Data on patients including age, clinical, laboratory, and histology were reviewed and analyzed to determine treatment outcome. *Results.* A total of 253 patients were studied. Their mean age was 69.11 ± 10.9 years (range 50–98). The most common symptoms at presentation included frequency 229 (90.5%) and poor stream 225 (88.9%). The most common complications at presentation were stones in 41 (16.2%) and bleeding in 37 (14.6%). The most common comorbid conditions were hypertension and diabetes found in 72 (28.5%) and 23 (9.1%), respectively. Transvesical prostatectomy was done for most of the patients, 126 (49.8%). Clot retention and wound infection were the commonest postoperative complications accounting for 19 (7.5%) each. Transient incontinence occurred in 17 (6.7%) patients. There was 1 (0.4%) mortality. *Conclusion.* Open prostatectomy still has a prime place in the operative treatment of BPH with acceptable postoperative morbidity and very low mortality in the developing world with no facilities for TURP.

## 1. Introduction 

Open prostatectomy has been the operative treatment for benign prostatic enlargement for several decades until the advent of transurethral resection of the prostate TURP (the gold standard) [1] and other state-of-the act modalities like transurethral interstitial LASER ablation [2], thermotherapy [3], and needle ablation [4]. This led to the gradual reduction in open prostatectomy in the developed world. In the developing countries, however, skills in traditional open surgery are mandatory, because the patients present late with very large prostates. A gland that is too large and completely obscuring the trigone and the ureteric orifices will not be comfortably resected transurethrally. Most urologists are comfortable with removing glands in the range of 50–75 g, transurethrally open surgery is, therefore, recommended for larger glands [5]. Comorbid medical conditions and complications of BPH at presentation are also indications for open prostatectomy [6]. The aim of this study was to review our ten-year experience with open prostatectomy, with special emphasis on the challenges of late presentation, complications of lower urinary tract obstruction, and comorbid medical conditions.

## 2. Patients and Methods

All patients who underwent open prostatectomy for BPH at the UMTH between January 2001 and December 2010 were retrospectively reviewed. Details of their biodata, clinical presentation, diagnostic investigations, operative treatment (Table 5), histological reports, postoperative complications, and other outcomes of surgery were extracted from their hospital records and analyzed. The diagnosis of BPH was made on clinical assessment, which included digital rectal examination and abdominopelvic ultrasound scan additional tests like PSA level and prostatic biopsy were reserved for suspicion of malignancy and when confirmed were excluded from the study. Open prostatectomy was performed on all the patients after resuscitation and initial urethral catheterization for continuous drainage where necessary, ensuring optimal renal function and treating any urinary tract infection with antibiotics based on sensitivity. Preoperative blood transfusion was given for bleeding prostates that came with anaemia. Methods of prostatectomy included transvesical, transcervical (low transvesical), and retropubic ones based on complications at presentation and surgeon's discretion. In low transvesical (transcervical) one, the incision is placed transversely across the bladder neck.

## 3. Results

This is a ten-year (January 2001–December 2010) retrospective review of open prostatectomy in 253 patients that had benign prostatic hyperplasia (BPH).

Table 1 depicts the age distribution of the patients. The mean age was 69.1 ± 10.9 years (range 50–98). The 70–79-year age group accounted for the highest number of patients (76), that is, 30.0%. 

The main symptoms at presentation as seen in Table 2 are frequency of micturition in 229 (90.5%) patients, poor urinary stream 225 (88.9%), and difficulty in passing urine 142 (56.1%). The mean duration of symptoms was 25.8 ± 27.5 months (range 1–168 months). All the patients had enlarged prostate with grossly benign features on digital rectal examination and abdominopelvic ultrasound scan. The complications at presentation (Table 3) included urinary tract stones in 41 (16.2%), groin hernia in 38 (15.0%); the least complication at presentation was impaired renal function in 18 (7.1%) patients. Vaginal hydrocele was an incidental finding in 12 (4.7%) of the patients.

A total of 38 (15%) patients were anaemic at presentation (i.e., PCV less than 30%). The urine culture was positive for bacteria in 135 (53.4%). This warranted antibiotic therapy in these patients. Clinically significant electrolyte derangement (renal impairment) was seen in 18 (7.1%). The premorbid conditions in the patients included hypertension in 72 (28.5%), diabetes 23 (9.1%), arthritis 17 (6.7%), and Parkinson's disease in 11 (4.3%) of the patients; lumbar spondylosis was seen in 9 (3.6%). Dementia 4 (1.6%) and 4 (1.6%) patients had various allergies while epilepsy was seen in 4 (1.6%) and HIV 5 (1.9%). Three (1.2%) were blind and 2 (0.8%) were deaf. Most of the patients had open prostatectomy under spinal anaesthesia 208 (82.2%), whereas general anaesthesia was used in 45 (17.8%) patients. Transvesical prostatectomy was done for most of the patients and accounted for 126 (49.8%), followed by retropubic in 104 (41.1%). Low transvesical was another method in 23 (9.1%).

Intraoperative findings were particularly for the prostate itself and the gross appearance of the bladder as depicted in Table 4. Majority of the patients 119 (47.0%) had bladder wall hypertrophy, while trabeculations and sacculation of the bladder were seen in 39 (15.4%). Diverticuli and bladder stones were seen in 26 (10.3%) and 35 (13.8%), respectively. The prostate was seen to have an isolated median lobe enlargement in 36 (14.2%) of the patients, global enlargement in 202 (79.8%), while 6 (2.4%) were found to be fibrotic. The mean estimated blood loss was 110 mls (85–238 mls). 

The postoperative complications (Table 6) included retrograde ejaculation in 20 (7.9%); clot retention and wound infection were seen in 19 (7.5%) each. The least postoperative complication was renal failure seen in 1 (0.4%) patient. 

The mean weight of the prostate was 103.6 ± 67.0 (range 36–426 g). The histology of removed prostates showed benign features (BPH) in 239 (94.5%) and malignant foci in background benign hyperplasia (incidental carcinoma) in 14 (5.5%) of patients. 

## 4. Discussion

Until 20–25 years ago, open surgery was the most common approach to the prostate in benign prostatic hyperplasia. From the late 1970s, however, the development of endoscopes led to a gradual reduction in the number of open surgical operations for the prostate, and open prostatectomy continued to decrease rapidly in favor of the minimally invasive endoscopic techniques. Therefore, open surgery for BPH is declining though still performed. Skills in traditional open surgery are still the main approach and are in fact mandatory in most developing countries who cannot afford widespread use of minimally invasive techniques of prostatectomy. Open surgery is still important to all surgeons because indications for it still exist.

 Two hundred and fifty-three patients, who had open prostatectomy for BPH, were reviewed indicating their clinical presentation, associated comorbid medical conditions, and management outcome. The mean age of 69.11 years is not different from other studies [7]. The clinical features of BPH are the same with other studies; however the complications at presentation are in sharp contrast to what is obtainable in developed countries [6, 8] where recurrent urinary retention predominates. Haemorrhage, very huge prostates, associated with diverticuli, bladder stones, and impaired renal function which are all features of late presentation are common in the developing countries [9, 10]. In addition to late presentation is the prevalence of comorbid medical conditions like hypertension and diabetics. The combination of the above factors is a major indication for open surgery, even where there are facilities for modern minimal access techniques. Massive persistent haemorrhage in BPH is a fairly common and life-threatening complication at presentation in this study; there were 37 (14.6%), lower than that reported by Ramyil et al. [11]. These patients were managed by emergency transvesical prostatectomy, which is a safe procedure that allows for direct control of haemorrhage. Other methods employed in dealing with bleeding prostates include continuous bladder irrigation, bladder instillations, and selective arterial prostatic embolization [12, 13]. 

Diverticuli, bladder stones, and hernias are common complications associated with lower urinary tract obstruction. These were dealt with during prostatectomy, the same primary surgery. Obstructive lower urinary tract symptoms complicated by impaired renal function evidenced by elevated creatinine are known to improve or return to normal after variable periods of continuous urethral catheter drainage. This was our experience in most of our patients who improved within 7 to 14 days. 

Comorbid medical conditions are common among this group of elderly males. This leads to an increased incidence of complications during or after prostatectomy; therefore, a prudent care is required to make them safe for surgery [6]. In this study, the most common comorbid medical conditions were diabetes and hypertension, which is similar to other studies [14].

Open prostatectomy is still safe though with longer hospital stay than minimally invasive techniques [15].

In this series, the mean weight of removed prostates was 103.6 ± 67.0 with a range of 36–426 g; this is far more than the weight recommended by EAU Guidelines for open prostatectomy which is between 80 and 100 g [16]. This is still a problem of late presentation in the developing countries. Open prostatectomy is accepted as the treatment of choice for large glands (>80–100 g). Three recent randomized controlled trials (RCTs) have shown that Holmium laser enucleation and PVP (transurethral photoselective vaporization of the prostate) have comparable outcomes to open prostatectomies in men with glands between 70 and 100 mls [17]. This underscores the fact that open prostatectomy though the most invasive is still the most effective and durable treatment. 

 Clot retention and wound infection were the major postoperative complications in this series comparable to similar studies [15, 18]. Transient incontinence, a well-known complication especially in transvesical prostatectomy, was comparable to other similar series [15, 18]; however, the incidence of retrograde ejaculation was low. This may be attributed to the cultural barrier or inhibition of volunteering such information [19]. The low mortality rate of 0.4% is in keeping with current trend of decreasing mortality from major urological procedures [20]. The one patient that died (0.4%) was as a result of pulmonary embolism following deep vein thrombosis, which is not an uncommon complication of major pelvic surgeries [21].

## 5. Conclusion

Open prostatectomy with its attendant lower mortality and morbidity is still necessary in the operative management of benign prostatic hyperplasia. Open procedures for BPH are still necessary for huge, bleeding, and complicated prostates despite the advent of modern minimally invasive techniques. For safe surgery and avoidance of postoperative complications, comorbid medical conditions associated with advancing age must be addressed.

## Figures and Tables

**Table 1:** Age distribution.

Age (yrs)	Number (%)
50–59	52 (20.6)
60–69	72 (28.5)
70–79	76 (30.0)
80–89	39 (15.4)
>90	14 (5.5)

Total	253 (100)

**Table 2:** Symptoms at presentation.

Symptoms	Number (%)
Frequency	229 (90.5)
Poor stream	225 (88.9)
Difficulty in passing urine	142 (56.1)
Acute urinary retention	124 (49.0)
Urgency/urge incontinence	116 (45.3)
Nocturia	109 (43.1)
Hesitancy	93 (36.8)
Incomplete emptying of bladder	80 (31.6)
Postmicturition dribbling	60 (23.7)
Haematuria	30 (11.9)
Incontinence	6 (2.4)

**Table 3:** Complications at presentation.

Complications	Frequency (%)
Stones	41 (16.2)
Hernias	38 (15.0)
Bleeding	37 (14.6)
Urinary tract infection	27 (10.7)
Hydronephrosis/hydroureters	21 (8.3)
Haemorrhoids	20 (7.9)
Renal impairment	18 (7.1)
Hydrocele (incidental finding)	12 (4.7)

**Table 4:** Intraoperative findings.

Intraoperative findings	Frequency (%)
(a) Bladder	
Thickened wall	119 (47.0)
Diverticuli	26 (10.3)
Trabeculations/sacculations	39 (15.4)
Stones	35 (13.8)
(b) Prostate	
Median lobe enlargement	36 (14.2)
Global enlargement	202 (79.8)
Fibrotic prostate	6 (2.4)

**Table 5:** Operative technique.

Operative technique	Frequency (%)
Transvesical	126 (49.8)
Low transvesical	23 (9.1)
Retropubic	104 (41.1)

Total	253 (100)

**Table 6:** Postoperative complications.

Complications	Frequency (%)
Retrograde ejaculation	20 (7.9)
Clot retention	19 (7.5)
Wound infection	19 (7.5)
Incontinence of urine	17 (6.7)
Erectile dysfunction	17 (6.7)
Urinary tract infection	14 (5.5)
Vesicocutaneous fistula	13 (5.1)
Others	27 (10.7)

Others:

Epididymoorchitis = 8, cardiac failure = 2, and renal failure=1.

Bladder neck stenosis/stricture = 5.

Irritable bladder syndrome = 5.

Pneumonia/atelectasis = 4.

Deep vein thrombosis = 2.
